# Prefrontal - subthalamic pathway supports action selection in a spatial working memory task

**DOI:** 10.1038/s41598-020-67185-1

**Published:** 2020-06-26

**Authors:** Carla Heikenfeld, Sara Mederos, Changwan Chen, Tatiana Korotkova, Alfons Schnitzler, Alexey Ponomarenko

**Affiliations:** 1 Institute of Clinical Neuroscience and Medical Psychology, Medical Faculty, Heinrich-Heine-University Düsseldorf, Düsseldorf, Germany; 2 Department of Functional and Systems Neurobiology, Instituto Cajal, CSIC, Madrid, Spain; 3 Max Planck Institute for Metabolism Research, Cologne, Germany; 4 Institute for Vegetative Physiology, Medical Faculty, University of Cologne, Cologne, Germany; 5 Institute of Physiology and Pathophysiology, Friedrich-Alexander-University Erlangen-Nürnberg, Erlangen, Germany

**Keywords:** Neural circuits, Spatial memory, Working memory, Neuroscience, Prefrontal cortex, Parkinson's disease

## Abstract

Subthalamic nucleus (STN) is the main source of feed-forward excitation in the basal ganglia and a main target of therapeutic deep brain stimulation in movement disorders. Alleviation of motor symptoms during STN stimulation can be accompanied by deterioration of abilities to quickly choose between conflicting alternatives. Cortical afferents to the subthalamic region (ST), comprising STN and zona incerta (ZI), include projections from the medial prefrontal cortex (mPFC), yet little is known about prefrontal-subthalamic coordination and its relevance for decision-making. Here we combined electrophysiological recordings with optogenetic manipulations of projections from mPFC to ST in mice as they performed a spatial working memory task (T-maze) or explored an elevated plus maze (anxiety test). We found that gamma oscillations (30–70 Hz) are coordinated between mPFC and ST at theta (5–10 Hz) and, less efficiently, at sub-theta (2–5 Hz) frequencies. An optogenetic detuning of the theta/gamma cross-frequency coupling between the regions into sub-theta range impaired performance in the T-maze, yet did not affect anxiety-related behaviors in the elevated plus maze. Both detuning and inhibition of the mPFC-ST pathway led to repeated incorrect choices in the T-maze. These effects were not associated with changes of anxiety and motor activity measures. Our findings suggest that action selection in a cognitively demanding task crucially involves theta rhythmic coordination of gamma oscillatory signaling in the prefrontal-subthalamic pathway.

## Introduction

Adaptive choices involve exquisite temporal coordination of cognitive, affective and motor processing. Studies in rodents and primates showed the role of basal ganglia in action selection via encoding and modulation of action outcomes in the striatum and globus pallidus^1,2^. These regions are interconnected with the subthalamic nucleus^2^, stimulation of which in Parkinson’s disease patients can impair rapid decision-making^3^. Responding to conflicting alternatives is believed to involve intermittent STN-driven inhibition of thalamocortical projections^4^. Action outcomes are also encoded in the mPFC^5^, integrity of which is required for correct decisions in a delayed non-matching-to-place task (T-maze)^6^. Extensive projections of mPFC to the subthalamic region (ST), including ventromedial aspects of the subthalamic nucleus (STN) and zona incerta (ZI), received considerable attention in functional models of basal ganglia circuits^7^, yet the significance of this pathway for decision-making has not being studied.

Compelling evidence implicates periodic changes of neuronal excitability during network oscillations in the coordination of neuronal activity across brain regions^8,9^. Gamma oscillations in mPFC support attention in goal-driven tasks^10^, they also occur in the basal ganglia during alertness, movement^11^ and somatosensory processing^12^. Oscillations in distinct sub-theta (<5 Hz) and theta (5–10 Hz) frequency bands coordinate timing of gamma oscillations (i.e. phase - amplitude coupling, PAC) in mPFC and other regions involved in goal-directed behaviours, including amygdala and hippocampus in a state-dependent way^13^. Pathological PAC of beta and fast oscillations in the basal ganglia leads to motor impairment^14^. An increased power of prefrontal and subthalamic slow oscillations in various decision-making paradigms^15–18^ is thought to assist prefrontal-subthalamic signaling, essential for adaptive adjustment of decision thresholds^19^. Decision-making in a spatial working memory task relies of gamma rhythmic signaling in circuits including mPFC^6,20^, yet functions of prefrontal-subthalamic PAC remain largely unknown.

Here, we asked whether PAC supports prefrontal-subthalamic coordination in the mouse and examined whether this signaling is involved in decision-making in cognitive vs. affective paradigms. Using electrophysiological recordings from the mouse ST and mPFC, theta/gamma PAC is identified as a dominant regime of interaction between these regions in a delayed non-matching-to-place (T-maze) task. In the mouse, extensive mPFC projections are found in both subthalamic subregions, ZI and STN. Optogenetic manipulations opposing theta/gamma PAC in the mPFC-ST pathway differentially affected decision-making in T- and elevated plus mazes.

## Methods

### Experimental subjects

Wild-type mice (C57BL/6) 12–25 weeks old were used. Mice were kept on a 12 h light/dark cycle. All animal experiments were conform with international and national guidelines and were approved by LaNuV Nordrhein-Westfalen, Germany.

### Virus injection

Mice were anaesthetized with isoflurane and placed in a stereotaxic frame (David Kopf Instruments). During the entire surgery reflexes and respiration were checked. For manipulations of the mPFC-ST-pathway mice were bilaterally injected in mPFC (AP: 1.5, ML: +/− 0.2, DV: 3.1–3.3 mm) with AAV-hSyn-NpHR-TS-p2A-hChR2(H134R)-EYFP (eNPAC 2.0, titre 2.9 × 10^13^ vg ml^−1^) virus provided by K. Deisseroth or a control AAV-hSyn-EYFP (3.3 × 10^12^ vg ml^−1^, UNC Vector Core). An assembly of a 27 Gauge, 0.4 × 0.25 mm bevelled metal needle and a 0.1 mm silica capillary tube was connected via a tube (Intramedic Clay Adams Brand, outer diameter 0.38 mm, inner diameter 1.09 mm) with a 5 µl microsyringe pump (PHD Ultra, Harvards Apparatus). Volume per injection site was 250 nl injected at 200 nl/min speed. After infusion the needle was kept at the injection site for 10 min.

### Stereotaxic implantations

Optic fibres (diameter 100 µm, 0.22 NA, Thorlabs) were glued to zirconia ferrules (Precision Fibre Products) and implanted in 9 mice above STN (bilaterally AP: −2, ML: +/− 1.6, DV: 4.22 mm and AP: −0.27, ML: 1.6, DV: 4.47 mm with an angle of 20°). Five further animals were implanted in mPFC and/or ST with multichannel arrays made of 45 µm formwar-insulated tungsten wire (California Fine Wire Company) using the following coordinates: mPFC (AP: 1.5 mm, ML: 0.3 mm, DV: 3.1 mm) and ST (AP: −1.9 mm, ML: 1.6 mm, DV: 4.6 mm). Ground and reference wires were connected to a screw placed in the skull above the cerebellum. Implants were secured with additional bone screws and dental acrylic.

### Data acquisition

Electrodes were connected to an operational amplifier (HS-8, Neuralynx) to eliminate cable movement artefacts. Electrophysiological signals were band-pass filtered (1 Hz − 10 kHz, Digital Lynx, Neuralynx) and acquired continuously at 32 kHz. A red LED was attached to the headset to track the animals’ position at 25 Hz. Timestamps of laser pulses were acquired together with electrophysiological data.

### Optogenetic stimulation

Fiberoptic patch cords with protective wrapping (Thorlabs) were connected to the implanted fibers. For unilateral optogenetic stimulation the patch cord was connected to a 473 nm diode-pumped solid-state laser (DPSS, Laserglow Technologies). Optogenetic stimulation of mPFC-ST projections in the right hemisphere consisted of seven 5 ms blue light pulses at 67 Hz repeated at 3 Hz. For bilateral inhibition the implanted fibers were connected via a dual patch cord to a 593 nm DPSS laser (Laserglow Technologies). For optogenetic inhibition continuous light pulses were applied during test trials in the T-maze or at the intersection of open and closed arms of the elevated plus maze. Light power output measured before each experiment with a power meter (PM100Dm, Thorlabs) was 8 to 20 mW (stimulation) and 15 mW (inhibition) from the tip of each patch cord, with the light transmission of optic fibre implants of 50%.

### T-Maze

T-maze was made of a dark-grey painted wood (start arm, 46 ×11 ×10 cm, choice arm, 80 ×11 ×10 cm). A spatial non-matching to place task was performed as described elsewhere^20^ and consisted of pairs of a sample and test trials. During the sample trial mice could run only to one segment of the choice arm since the opposite segment (chosen pseudorandomly, keeping number of left and right turns per session equal) was closed with a door of the same colour and material as the maze. A reward (drop of diluted condensed milk) was placed at the end of the arm. Sample and test trials were separated by a delay of 20 sec, during this time the animal was placed in a chamber at the entrance to the start arm. During the test trial both segments of the choice arm were open. Alternation was rewarded by placing condensed milk in the end of the previously unvisited segment of the choice arm. Mice ran one trial at a time with inter-trial intervals of 5 min. In total each animal ran 50 sessions during 5 consecutive days, 10 trials per day. The performance was evaluated for blocks of 20 trials to account for possible differences during the task acquisition. Optogenetic stimulation (trials 1–40) or inhibition (trials 41–50) was applied from the beginning (entrance to the start arm opened) till the end of each test trial.

### Elevated plus maze

The elevated plus maze test was performed as described elsewhere^21^. The enclosure had two closed and two open arms (30 ×10 ×5 cm each), painted in dark-grey and was placed one meter above the ground. At the beginning of a 5 min session the animal was placed at the intersection of the arms, with the head towards an open arm. Two sessions, one for optogenetic inhibition and one for stimulation, 3 days apart were performed for each animal. Light was delivered as the mice entered the square with two paws and stopped when all four paws were out of the square as they entered an open or closed arm.

### Histology and microscopy

After completion of experiments, the brains were fixed for 24 hours in 4% paraformaldehyde, 1% PBS and cut in 50 µm slices with a vibrotome (EMS 4500, Electron Microscopy Science). Images were taken with Leica, TCS SP8 and Zeiss, Imager 2 microscopes.

### Analysis of electrophysiological data

LFP was obtained by down-sampling of the wide-band signal to 1250 Hz using Neurophysiological Data Manager^22^. Cross-frequency phase-amplitude coupling (PAC) was analysed similar to^23^. Theta and sub-theta oscillatory epochs were detected based on power ratio of 3 between 5–10 and 2–5 Hz frequency bands. Power spectra were computed using the multitapper method. Phase was computed for signals band-pass filtered in 5–10 Hz and 2–5 Hz bands using Hilbert transform. The signal was then filtered in the gamma (30–70 Hz) band, oscillation peaks were detected, their amplitudes were obtained and theta and sub-theta phases assigned. Oscillation cycles were divided in 15 phase bins, the amplitude of gamma oscillations was averaged for each bin. To study cross-regional PAC, amplitudes of gamma oscillations and phases of slow (theta and sub-theta) oscillations were computed for mPFC and ST signals, respectively. PAC modulation coefficient was computed from amplitude-phase histograms similar to^23^ by: Q = r (G_max_ − G_min_)/(G_max_ + G_min_), where G_max_ and G_min_ are the maximal and minimal gamma peak amplitudes within the slow oscillations cycle; r, coefficient of determination for the fitted sine function.

### Statistical analysis

Statistical tests were chosen according to the experimental design. Two-tailed t-test or Mann-Whitney test were used for two-group comparisons depending on the normality of the distribution. For comparisons between behaviours (chamber vs. test runs in the T-maze), theta PAC was assessed with analysis of covariance using theta rhythm amplitude as a covariate as described elsewhere^23^. Descriptive statistics are reported as mean ± SEM.

## Results

**Figure 1:**
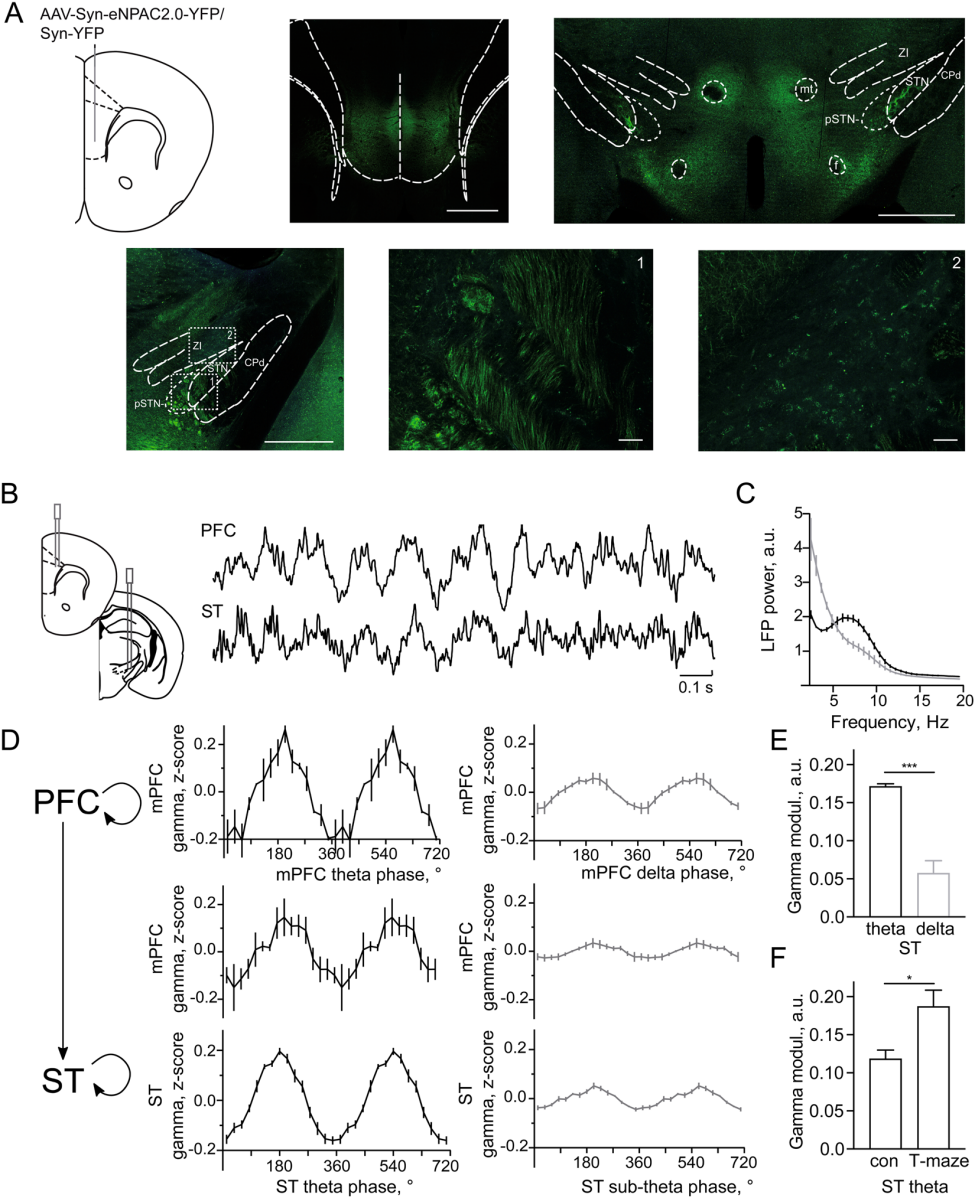
Prefrontal-subthalamic connectivity and rhythmic coordination. (**A**) Example confocal images showing projections of mPFC to ST (two of 3 mice). Scale bars: 750 µm (top left), 1000 µm (top right), 500 µm (bottom left), 50 µm (bottom 1, 2). (**B**) LFP signals during theta rhythmic epochs showing concurrent gamma oscillations. (**C**) Power spectral density of LFP recorded in ST during theta (black line) and sub-theta (grey line) epochs (n = 5 mice). (**D**) Phase-amplitude coupling (PAC) of theta and gamma oscillations vs. sub-theta and gamma oscillations in mPFC (upper panels), between mPFC and ST (middle panels, n = 3 mice) and in ST (lower panels, n = 5 mice). (**E**) PAC magnitude during theta and sub-theta oscillations in ST (p < 0.0001, t-test). (**F**). PAC magnitude during theta oscillations in ST in the chamber between runs (con) and during correct runs in the T-maze (p < 0.05, t-test). Data are presented as mean ± SEM.

To visualize and manipulate projections from mPFC to ST we introduced an AAV-hSyn-eNPAC2.0-EYFP virus^20^ to mPFC of wild-type mice. Confocal imaging revealed distinct bundles of mPFC-originating fibres leaving the cerebral peduncle close to the ventromedial part of STN and the adjacent parasubthalamic nucleus, and arborizing in these regions and dorsally in ZI (Fig. 1A). These findings are in line with previous reports of mPFC efferents to STN established using chemical tracers in primates and rats^24^ and to ZI, using retrograde viral tracing in mice^25^.

Next we studied the coordination of network oscillations in mPFC and ST using local field potential (LFP) recordings, while implanted mice were trained in the delayed non-matching-to-place (T-maze) paradigm. LFP signals recorded in both regions featured epochs with a leading rhythmicity either in sub-theta (2–5 Hz) or in theta (5–10 Hz) bands (Fig. 1B,C). The amplitude of gamma (30–70 Hz) oscillations in mPFC periodically changed according to the phase of concurrent slower oscillations, in agreement with previous reports of PAC in mPFC (Fig. 1D^26^). The LFP in ST also displayed gamma oscillations modulated by locally recorded sub-theta and theta rhythms (Fig. 1D). Surprisingly, PAC within mPFC and ST was accompanied by PAC across the regions, indicated by changes of the mPFC gamma oscillations amplitude according to the phase of ST sub-theta and theta oscillations (Fig. 1D).

While the changes of the gamma amplitude were coordinated between the two regions during both slow rhythms, the magnitude of PAC was frequency-dependent, being several-fold higher during theta than during sub-theta oscillatory epochs (Fig. 1E), showing a similar pattern across behaviours (theta/sub-theta PAC: 3.8 ± 0.9, in the chamber where a mouse was located between runs, vs. 4.3 ± 0.8 in the T-maze, p = 0.4, t-test). Furthermore, the magnitude of PAC during theta oscillations was substantially higher in the T-maze than in the chamber (Fig. 1F).

**Figure 2:**
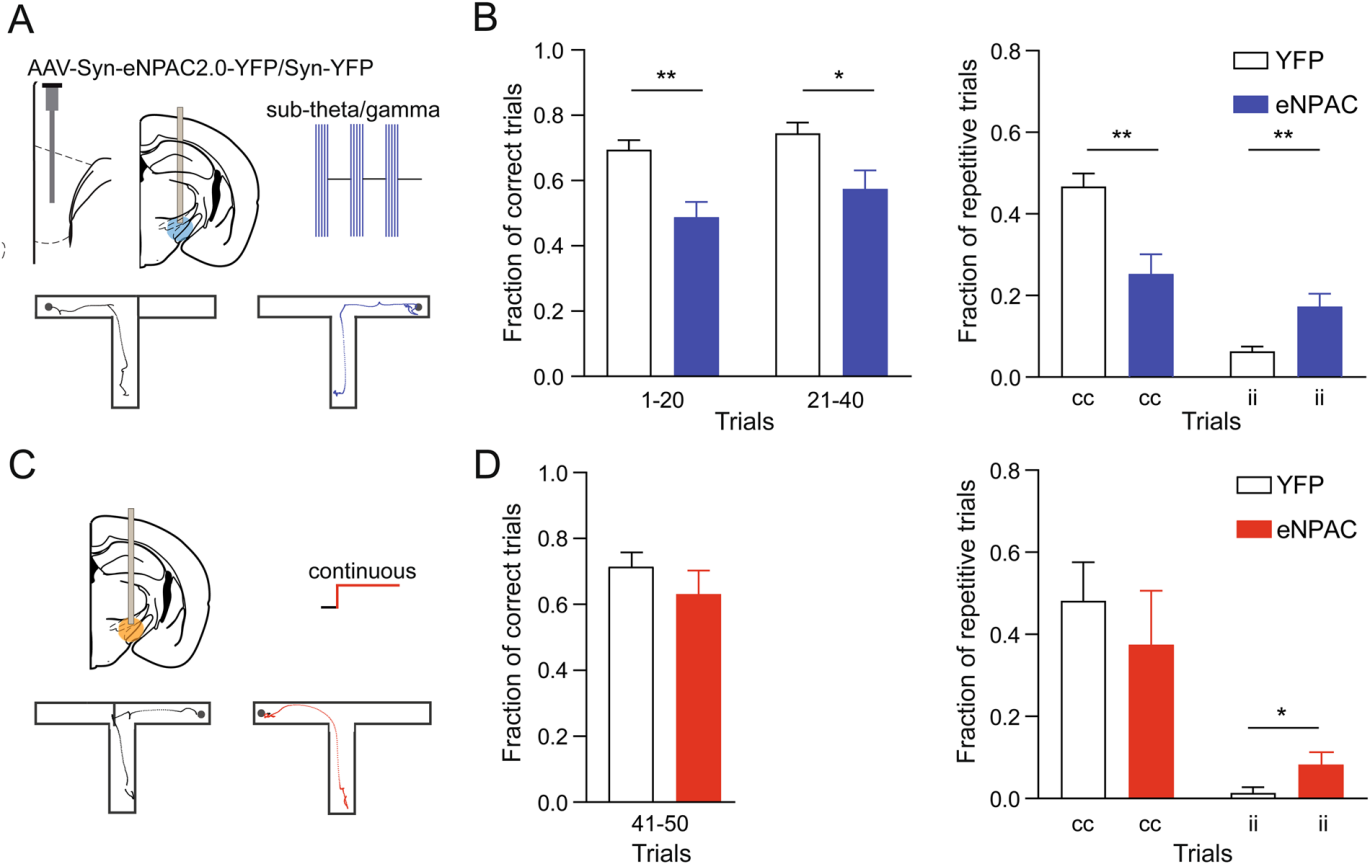
Optogenetic manipulations of mPFC-ST projections impair performance in a non-matching-to-place task. (**A**) Experimental design, optogenetic cross-frequency theta -> sub-theta detuning stimulation. (**B**) Left panel, number of correct choices in T-maze was reduced during the mPFC-ST detuning stimulation (trial 1–20: YFP: N = 9 mice, eNPAC: N = 8 mice; p < 0.01, t-test; trials 21–40: YFP: N = 9 mice, eNPAC: N = 8 mice, p < 0.05). Right panel, mPFC-ST detuning stimulation reduced fraction of repeated correct trials (cc, YFP: N = 9 mice, eNPAC: N = 8 mice, p < 0.01) and increased fraction of repeated incorrect trials (ii,YFP: N = 9 mice, eNPAC: N = 8 mice, p < 0.01). (**C**) Experimental design, optogenetic inhibition of the mPFC-ST pathway. (**D**) Number of correct trials (left panel, trial 51–60: YFP: N = 9 mice, eNPAC: N = 8 mice; p = 0.4) and repeated correct (cc) and incorrect (ii) trials (right panel) during optogenetic inhibition (cc, p = 0.5; ii, p < 0.05). Data are presented as mean ± SEM.

To investigate behavioural functions of the rhythmic signaling via mPFC to ST projections in the T-maze we optogenetically detuned PAC in the mPFC-ST pathway. To do that, we opposed a more efficient in the T-maze theta/gamma coordination by the subtheta/gamma stimulation, mimicking a less efficient during this behavior PAC (Fig. 2A). Similar projection-specific manipulations in other brain regions entrained neuronal discharge and network oscillations^20,23^. During the optogenetic detuning of the mPFC-ST pathway the number of correct trials decreased in opsin-expressing mice compared to control YFP-expressing mice (Fig. 2B).

Moreover, the stimulation impaired temporal stability of the performance, reducing fraction of repeated correct trials and increased fraction of repeated incorrect trials (Fig. 2B). The latter effect was induced also by an optogenetic NpHR-inhibition of mPFC-ST projections applied in eNPAC2.0 and control YFP-expressing mice during an additional block of trials, i.e. in well trained mice (Fig. 2C,D). These results suggest that the mPFC-ST pathway is crucial for action selection not only during learning but also when the task is already acquired.

**Figure 3:**
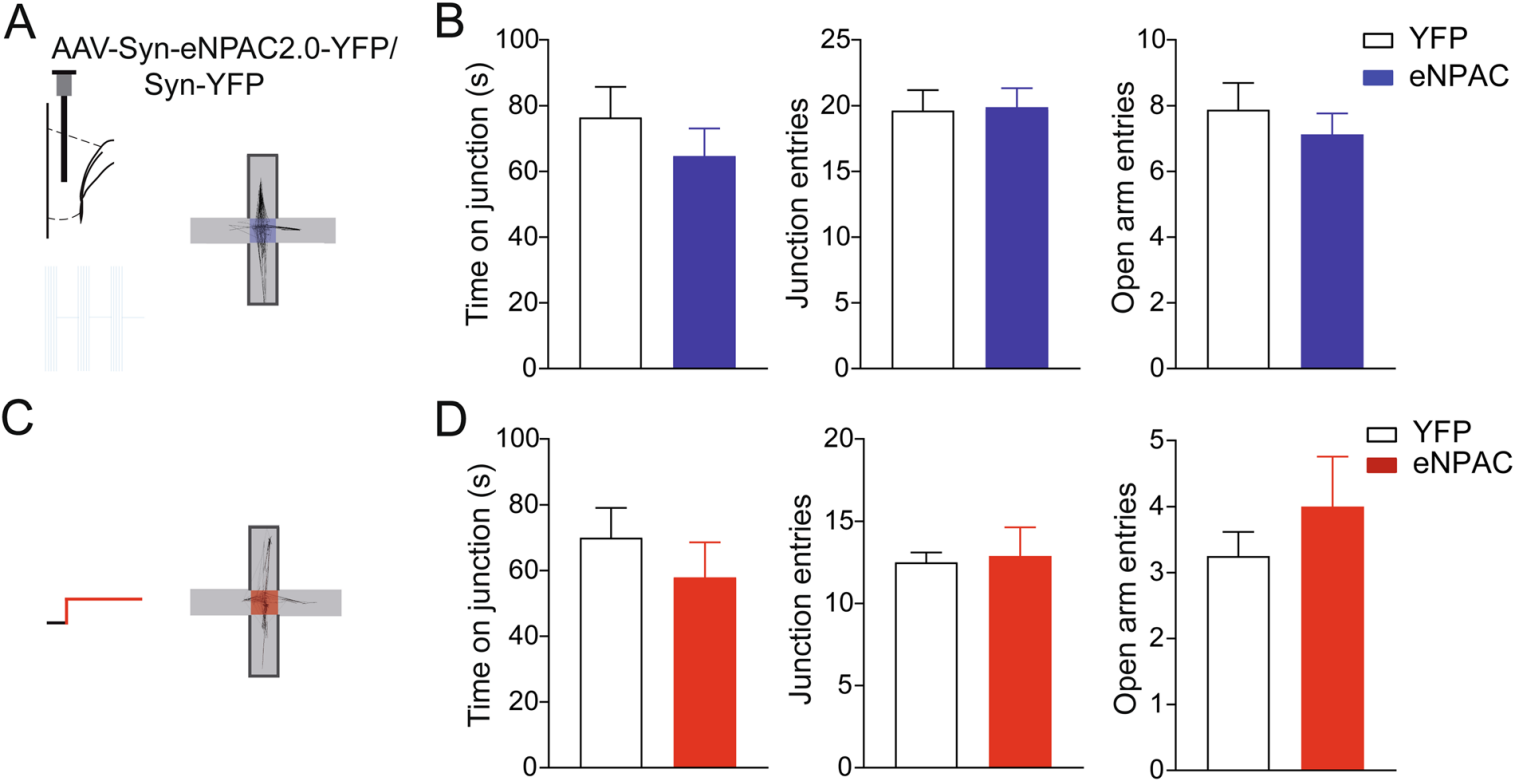
Intact behaviour in the elevated plus-maze during manipulations with the mPFC-ST pathway. (**A**) Experimental design, cross-frequency theta -> sub-theta detuning optogentic stimulation. Light was applied at the maze junction. (**B**) Time spend in the decision zone, (left panel, time on junction, p = 0.4, Mann-Whitney test), measures of activity (middle panel, junction entries, YFP: N = 8 mice, eNPAC: N = 8 mice, p = 0.9, t-test) and anxiety (right panel, entries to open arms, p = 0.4). (**C**) Experimental design, continuous optogentic inhibition at the maze junction. (**D**) Time on junction (p = 0.2, Mann-Whitney test) and other behavioral measures were not changed (p = 0.8 and p = 0.4 for left and right panels, respectively, t-test). Data are presented as mean ± SEM.

Choices in the T-maze rely on spatial working memory but can be influenced by changes of motor activity and potential effects of the stimulation on the processing of emotional valence of choices in the task. To address these possibilities and study effects of manipulations with the mPFC-ST pathway on anxiety vs. novelty-seeking driven action selection we performed optogenetic detuning of prefrontal projections in the elevated plus maze in a behavior-dependent closed-loop protocol, when the mouse entered the junction of open and closed arms (Fig. 3). During optogenetic stimulation the number of entries in open arms as well as the number of crossings between arms was similar between opsin-expressing and control groups (Fig. 3A,B), indicating that the stimulation neither changed motor activity nor modulated anxiety.

Opsin-expressing and control mice showed no differences in the number of left and right turns to open arms (right/left ratio, averaged between exits from each closed arm: p > 0.05 for each group, one-sample t-test vs. ratio 1; between groups, p > 0.05, t-test). Similarly, optogenetic inhibition of the mPFC-ST pathway did not affect measures of decision-making and anxiety in the elevated plus maze (Fig. 3C,D). Neither optogenetic manipulation in the elevated plus maze led to overt changes of ongoing behaviour.

## Discussion

We found that prefrontal cortex and subthalamic region, connected in mice by a descending pathway, are coordinated by fast (gamma band) oscillations modulated preferentially at theta frequencies. Opposing the theta/gamma coupling by a sub-theta/gamma optogenetic stimulation of mPFC-ST projections, or by their inhibition, impaired the performance in a spatial working memory guided decision-making in T-maze while did not affect anxiety vs. novelty-guided decisions in the elevated plus maze. These results provide a projection-specific interventional evidence for the involvement of prefrontal inputs to ST in cognitive control and action selection proposed in recent electrophysiological, clinical and modeling studies^3,27–29^. Our study points to an upstream circuitry, network dynamics and different behavioral contexts acting in concert with mechanisms of suppressive responses, recently investigated using optogenetic manipulations of the ST output^30^.

Prefrontal afferents to ST have been characterized in primates and rats^31–33^. In primates, inputs from associative and limbic PFC to STN overlap more with each other and with motor afferents than do largely segregated cortico-striatal connections, suggesting a potential integration of this information streams at the level of STN. Extensive projections from infralimbic and prelimbic cortex to ZI have been recently shown in the mouse^25^, yet the long-standing question of their role in action selection remained unexplored using projection-specific tools. The STN and ZI in the rat display similar topography of cortical inputs suggesting a possible functional coordination of these two regions^33^, in particular regarding their influence on thalamocortical signaling^34–36^. Our projection imaging highlights similarities of mPFC-ST connectivity in mice with that in rats and non-human primates, showing a dorsoventral gradient of mPFC efferents^37,38^ with prelimbic and infralimbic cortex targeting ZI and the ventrolateral STN, regions involved in autonomic regulations and affective processes.

Compelling evidence supports the role of mPFC in the contextual control of action^39^. Performance in non-matching-to-place tasks requires functionally intact mPFC^40–42^ which signals antecedent choices^6,43^. These representations of the goal location during the sample (encoding) part of the task rely on inputs from the ventral hippocampus, optogenetic manipulations of which^6^, as well as of the prefrontal-thalamo-hippocampal circuit^43,44^, impair performance in the T-maze. Trajectory representations in this circuit depend also on ascending subcortical connections, as has been recently shown for the supramammillary nucleus^43^. Based on discharge phase correlations and LFP coherence, this and earlier studies suggested that memory-guided choices can be supported by the theta rhythmic coordination between mPFC and dorsal hippocampus^45–47^ and by the gamma oscillatory coordination between mPFC and ventral hippocampus^6^. Correct choices dependent on working memory can involve interactions of mPFC with the secondary motor cortex (M2), a rodent homolog of the crucial for proactive behavioural switching primate pre-supplementary motor area^48^. Furthermore, since a population of STN neurons also displays switch-selective activity^49^, neural representations of working memory and response inhibition from mPFC and M2, respectively, could be integrated within STN.

Contextual recall of remote memories and brief retention of recent experiences allow mPFC to adaptively bias behavioural choices, these functions involve recently studied projections of the ventromedial mPFC to various cortical and subcortical regions. Apart from the discussed above role of the connectivity with the hippocampus, choices to act, albeit in a more challenging setting of despair, are supported by projections to nucleus raphe^50^. Correct decisions in the identical to the applied here paradigm with various types of reward, involve gamma–frequency synchronization between mPFC and the lateral septum^20^. Projections to NAC, depending on input populations, signal aversion^51^ and reward^2,52^. A capacity of mPFC to retain information over short time intervals apparently supports a top-down prefrontal control over motor cortex during maintenance of action sets^53^. Persistence of maladaptive sets by frontal-subthalamic loops can lead to increased incidence of repeated incorrect trials during manipulations of mPFC-ST pathway. Accordingly, prefrontal and motor components of the hyperdirect pathway to STN are thought to subserve distinct functions in different behavioral contexts^19^, changes of the activity of these pathways is a primary action mechanism of the therapeutic deep brain stimulation^54^.

Decisions between conflicting alternatives are associated with an elevated theta-band power in the prefrontal cortex and STN across experimental paradigms^15,17,18,55,56^ with the mPFC, where conflict is initially detected^57^, leading STN theta according to a recent analysis using Granger causality^3^. Conversely, a large body of evidence indicates the involvement of beta oscillations in the STN during action stopping^49,58–62^. Our projection-specific manipulations support this dynamic perspective, which points to the mPFC-STN pathway as a part of the circuitry synchronized during conflict in the theta band and during stopping action in the beta band to oppose decisions driven by low magnitude corticostrital inputs^63^.

Cross-frequency PAC assessed in our study involves three frequency bands, with the amplitude of gamma oscillations changing in a quasi-sinusoidal fashion according to changes of excitability at slow, theta or sub-theta frequencies. While the present study focussed on LFP rather than on unitary recordings, multiple reports revealed entrainment of neuronal discharge by gamma oscillations coupled to slow rhythms in cortical and subcortical regions^20,64^. Modelling studies suggest that phases of coupling between the rhythms are determined by an optimal excitatory drive in the network, with too high or too low excitability resulting in quenching of the fast oscillation^65^. In the mPFC-ST circuit, excitation originates from the activity of cortical pyramidal cells, entrained by local gamma, theta and delta oscillations^9,66^, and probably modulated by feed-forward excitation and inhibition within STN and ZI, respectively, providing a mechanism for interaction between cortical inputs. Thus, while gamma oscillations in mPFC and ST can differ in the organization of their network generators, precise covariance of gamma amplitudes across regions provides an opportunity for integration of different subthalamic afferents. A concurrent increase in firing probabilities during PAC-synchronized gamma oscillations in different frontal areas (including secondary motor and prelimbic cortices) projecting to STN could be translated, by a coincidence detection mechanism, into a higher magnitude efferent signal leading to inhibition of incorrect responses. This scenario reconciles well with a higher power of theta oscillation during response inhibition^15,17,18,55,56^. Long-range coupling by gamma oscillations has been described between prefrontal and visual cortex during attention^67^, between prefrontal cortex and the lateral septum during decision making in the T-maze^20^ and, in a PAC regime, between hippocampus and striatum^68^. Altogether, our study underscores a crucial role of the prefrontal-subthalamic pathway in a wider circuitry, coordinated by coupled theta and gamma oscillations^6,46^, which supports working memory dependent decision-making.
